# Uricosuric targets of tranilast

**DOI:** 10.1002/prp2.291

**Published:** 2017-02-06

**Authors:** Asim K. Mandal, Adriana Mercado, Andria Foster, Kambiz Zandi‐Nejad, David B. Mount

**Affiliations:** ^1^ Renal DivisionsVA Boston Healthcare System and Brigham and Women's HospitalBostonMassachusetts; ^2^ Renal DivisionsDepartamento de NefrologíaInstituto Nacional de Cardiología Ignacio ChávezMexico CityMexico; ^3^ Renal DivisionBeth Israel Deaconess Medical CenterBostonMassachusetts

**Keywords:** Benzbromarone, GLUT9, gout, hyperuricemia, tranilast, URAT1, uric acid

## Abstract

Uric acid, generated from the metabolism of purines, has both proven and emerging roles in human disease. Serum uric acid in humans is determined by production and by the net balance of reabsorption and secretion in kidney and intestine. In the human kidney, epithelial reabsorption dominates over secretion, such that in normal subjects there is at least 90% net reabsorption of filtered urate resulting in a fractional excretion of <10%. Tranilast, an anti‐inflammatory drug with pleiotropic effects*,* has a marked hypouricemic, uricosuric effect in humans. We report here that tranilast is a potent inhibitor of [^14^C]‐urate transport mediated by the major reabsorptive urate transporters (URAT1, GLUT9, OAT4, and OAT10) in *Xenopus* oocytes; this provides an unequivocal molecular mechanism for the drug's uricosuric effect. Tranilast was found to inhibit urate transport mediated by URAT1 and GLUT9 in a fully reversible and noncompetitive (mixed) manner. In addition, tranilast inhibits the secretory urate transporters NPT1, OAT1, and OAT3 without affecting the secretory efflux pump ABCG2. Notably, while benzbromarone and probenecid inhibited urate as well as nicotinate transport, tranilast inhibited the urate transport function of URAT1, GLUT9, OAT4, OAT10, and NPT1, without significantly affecting nicotinate transport mediated by SMCT1 (IC_50_ ~1.1 mmol/L), SMCT2 (IC_50_ ~1.0 mmol/L), and URAT1 (IC_50_ ~178 *μ*mol/L). In summary, tranilast causes uricosuria by inhibiting all the major reabsorptive urate transporters, selectively affecting urate over nicotinate transport. These data have implications for the treatment of hyperuricemia and gout, the pharmacology of tranilast, and the structure‐function analysis of urate transport.

**Abbreviations**
ABCG2: ATP‐binding cassette subfamily G, member 2
AD: Alzheimer's disease
BHB: *β*‐hydroxy butyrate
Gluc: Gluconate
GLUT: Glucose transporter
Lac: Lactate
MRP4: multidrug resistance protein 4
MS: Multiple sclerosis
MSU: Monosodium urate
Nico: Nicotinate
NPT: Sodium phosphate transporter
OAT: Organic anion transporter
PAH: para‐aminohippurate
PD: Parkinson's Disease
PZA: Pyrazinoic acid
Sal: Salicylate
SLC: solute carrier gene
SMCT: Na^+^‐dependent monocarboxylate transporters
SUA: Serum uric acid
URAT1: Urate transporter‐1
*α*‐KG: *α*‐ketoglutarate

## Introduction

The causative link between hyperuricemia and disease is well established for gout, a common and excruciatingly painful inflammatory arthritis (Choi et al. 2005). Hyperuricemia has also been linked to the pathogenesis of gout‐associated comorbidities, including metabolic syndrome, hypertension (Perlstein et al. 2006; Feig et al. 2008; Forman et al. 2009), and diabetic nephropathy (Doria and Krolewski 2011). Hyperuricemia has an opposite, protective effect in neurodegenerative disease, including Parkinson's Disease (PD), multiple sclerosis (MS) (Hooper et al. 1998), and Alzheimer's disease/dementia (Spitsin and Koprowski 2010). Higher circulating uric acid levels thus reduce the risk of PD (Weisskopf et al. 2007) and the rate of disease progression (Ascherio et al. 2009).

In humans, 70–80% of serum urate is excreted in urine and the remaining 20–30% is secreted by the intestine. In the kidney, reabsorptive and secretory transporters maintain the balance between proximal tubular reabsorption and secretion (Mandal and Mount 2015). Approximately 90% of the urate filtered by renal glomeruli is reabsorbed into the blood by transepithelial transport in the renal proximal tubule, with ~10% fractional excretion; reabsorption thus dominates over secretion in the human kidney. Urate reabsorption in the proximal tubule involves the coordinated activity of several transporters. Sodium‐dependent reabsorption of organic monocarboxylates by apical Na^+^‐dependent monocarboxylate transporters SMCT1 and SMCT2, encoded by the *SLC5A8* and *SLC5A12* genes, respectively (Coady et al. 2004; Srinivas et al. 2005), increases the intracellular concentration of monocarboxylate anions that can then exchange with luminal urate via urate‐anion exchangers. Increases in the circulating concentrations of the SMCT substrates nicotinate, pyrazinoate, lactate, and ketones result in hyperuricemia (Gibson and Doisy 1923; Shapiro and Hyde 1957; Goldfinger et al. 1965; Gershon and Fox 1974), due to increased apical uptake of these filtered anions, increased intracellular concentrations in proximal tubular cells, and augmented urate‐anion exchange (Guggino and Aronson 1985). URAT1, encoded by *SLC22A12* gene, is the dominant apical urate/anion exchanger in humans; loss‐of‐function mutations in *SLC22A12* are associated with hypouricemia and hyperuricosuria. The “orphan” organic anion transporter (OAT) “ORCTL3″ (OAT10) has also been shown to mediate urate‐nicotinate exchange (Bahn et al. 2008). In addition, providing further heterogeneity, human OAT4 reportedly functions as an apical urate‐anion exchanger (Hagos et al. 2007), exchanging urate with divalent organic anions.

Multiple genome‐wide association studies (GWAS) have implicated variability in the *SLC2A9* (solute carrier gene family‐2, member 9) gene that encodes GLUT9 (glucose transporter 9) in determining serum urate concentration (SUA) (Doring et al. 2008; Vitart et al. 2008; Wallace et al. 2008). GLUT9 mediates urate exit at the basolateral membrane during reabsorption of urate in the proximal tubule. Separate basolateral urate transporters, OAT1 (organic anion transporter 1) and OAT3, encoded by *SLC22A6* and *SLC22A8*, respectively, function in urate secretion (Eraly et al. 2008)*,* transporting urate from blood into proximal tubular cells for secretion at the apical membrane. Urate secretion at the apical membrane is mediated by ATP‐driven efflux pumps MRP4 (multi‐drug resistance protein 4) (Van Aubel et al. 2004) or ABCG2 (Matsuo et al. 2009; Woodward et al. 2009) and/or electrogenic apical urate transporters NPT1/Oatv1 (encoded by the *SLC17A1* gene) (Jutabha et al. 2003; Iharada et al. 2010) and NPT4 (encoded by the *SLC17A3* gene) (Jutabha et al. 2010).

Uricosuric drugs such as benzbromarone (Enomoto et al. 2002), probenecid (Enomoto et al. 2002), fenofibrate (Uetake et al. 2010), lesinurad (Fleischmann et al. 2014), and losartan (Iwanaga et al. 2007) have been shown to inhibit URAT1, without assessing the effects on the entire panel of reabsorptive urate transporters. Antiuricosuric agents (e.g., nicotinate, pyrazinoate) can serve as the exchanging anion from inside tubule cells, thereby enhancing urate transport by URAT1 and OAT10 through trans‐stimulation (Guggino and Aronson 1985; Mandal and Mount 2015); at higher concentrations, these anions can also be uricosuric, due to extracellular cis‐inhibition at the apical membrane. Notably, although literature comparisons can certainly be made, there has been no comprehensive study of the interactions between specific uricosurics and anti‐uricosurics with all the various reabsorptive and secretory urate transporters.

Urate‐lowering therapy is a mainstay in the management of gout. Currently available urate‐lowering drugs in the U.S. include allopurinol, a purine analog that inhibits the enzyme xanthine oxidase; probenecid, a urate transport inhibitor; and febuxostat, a nonpurine inhibitor of xanthine oxidase. A substantial fraction of patients with gout fail to achieve adequate urate lowering with the current available drugs, indicating a need for alternative medications. Tranilast [N‐(3,4‐dimethoxycinnamoyl) anthranilic acid], an effective anti‐allergic drug developed in Japan, has been widely used for more than 40 years in Asia for the clinical treatment of bronchial asthma, atopic rhinitis, atopic dermatitis, and keloids. Tranilast causes potent reduction in SUA in healthy human subjects, at least partially due to uricosuric effects (Sundy and Kitt 2010). It also reportedly suppresses inflammation induced by monosodium urate (MSU) crystals in vivo (Serafini and Emerling 2010), with potential dual utility for “flare prophylaxis” (Borstad et al. 2004) during urate reduction.

This study was initiated to clarify the molecular mechanisms of the uricosuric effect of tranilast. A perceived limitation of other reports regarding uricosuric agents was the exclusive focus on URAT1, hence we studied the interaction of tranilast with all the reabsorptive urate transporters, in addition to a representative subset of secretory transporters. A secondary goal afforded by this approach was thus an across‐the‐board comparison of transport characteristics for multiple urate transporters, studied contemporaneously in the same expression system.

## Materials and Methods

### Complementary RNA (cRNA) expression in *Xenopus* oocytes

Studies in animals have been carried out in accordance with the Guide for the Care and Use of Laboratory Animals as adopted and promulgated by the U.S. National Institutes of Health, and were approved by the Institution's Animal Care and use Committee or local equivalent. Mature female *Xenopus laevis* frogs (NASCO, Fort Atkinson, MI) were subjected to partial ovariectomy under tricane (Ethyl 3‐aminobenzoatemethanesulfonate, SIGMA St Louis, MO) anesthesia (0.17% for 15–20 min) as described previously (Mount et al. 1999). In brief, a small incision was made in the abdomen and a lobe of ovary was removed. Subsequently, the oocytes were prewashed for 5 min in Ca^2+^‐free ND96 medium (96 mmol/L NaCl, 2 mmol/L KCl, 1 mmol/L MgCl_2_, and 5 mmol/L HEPES, pH 7.4) to remove blood and damaged tissue. Oocytes were then defolliculated by treatment with 3.5 mg/mL collagenase A (Roche, Indianapolis, IN) in Ca^2+^‐free ND96 medium for 60–70 min with gentle agitation at room temperature (25°C). After this treatment, oocytes were washed four times in Ca^2+^‐free ND96 medium, and then incubated in isotonic Ca^2+^‐containing ND96 medium (96 mmol/L NaCl, 2.0 mmol/L KCl, 1.8 mmol/L CaCl_2_, 1.0 mmol/L MgCl_2_ and 5 mmol/L Hepes, pH 7.4) containing 2.5 mmol/L pyruvate, and gentamycin (10 *μ*g/mL).

The indicated full‐length cDNAs were subcloned into the pGEMHE oocyte expression plasmid, 3′ of a T7 promoter and flanked by *Xenopus laevis‐*specific 5′‐ and 3′‐UTRs. Plasmid DNA was linearized at the 3′‐end of cDNAs by *Not1*,* Nhe1*, or *EcoR1* digestion, and cRNA was in vitro transcribed by using T7 RNA polymerase (mMESSAGE mMACHINE; Ambion, Austin, TX) following the suppliers protocol. Isopropanol‐precipitated, in vitro transcribed capped cRNA was washed with 70% ethanol, dried and then dissolved in sterile nuclease‐free water. The yield and RNA integrity of the capped cRNA samples were assessed by spectroscopy (at 260 nm) and 1% agarose‐formaldehyde gel electrophoresis, respectively. All cRNA samples were stored frozen in aliquots at −80°C until used. About 18 h after isolation, oocytes were injected with 50 nL of sterile water, 50 mmol/L tris pH 7.4, or 50 nL of a cRNA solution in 50 mmol/L tris buffer (pH 7.4) containing 25 ng of the indicated cRNA using fine‐tipped micropipettes by a microinjector (World Precision Instrument Inc. Sarasota, FL). The injected oocytes were incubated in isotonic ND96 medium (pH 7.4) containing 1.8 mmol/L CaCl_2_, 2.5 mmol/L pyruvate, and gentamycin (10 *μ*g/mL) at 16–18°C for approximately 48 h to allow expression of protein from injected cRNA.

### Western blotting

Total cellular protein for western blot analysis was prepared from groups of ~100 *X. laevis* oocytes injected with relevant cRNAs that were transcribed in vitro from related constructs. After 48 h of expression of protein from the injected cRNA, oocytes were transferred to 1.7 mL polypropylene microfuge tubes on ice and were lysed using a Teflon homogenizer in lysis buffer (50 mmol/L Tris‐HCl, pH 7.5, 50 mmol/L NaCl, 1 mmol/L EDTA, pH 8, 1% Triton X‐100) supplemented with protease inhibitors cocktail (Roche, Indianapolis, IN). After clearing the lysate off yolk and cellular debris by centrifugation at 2665 g for 10 min, the supernatant was stored at −80°C. Western blotting was performed using affinity‐purified rabbit polyclonal anti‐SLC2A9/GLUT9, anti‐URAT1, or anti‐OAT10 antibodies (MBL; Medical & Biological Laboratories Co. Ltd.) at a titer of 1:1000. Total lysates of proteins were fractionated using 7.5% SDS/PAGE gel electrophoresis (BIO‐RAD, Hercules, CA). Proteins were transferred to polyvinylidene difluoride (PVDF) membrane (BIO‐RAD) at 100 V for 3 h. The membrane was blocked in 5% nonfat dried milk in TBST. Primary antibodies were diluted in 5% milk in TBST and incubated with the membrane at room temperature for 2 h with continuous gentle shaking. Blots were washed in TBST and probed with an HRP‐conjugated secondary antibody (BIO‐RAD) in TBST containing 5% fat‐free milk for 1 h at RT. The membrane was then washed four times with TBST and chemiluminescence performed using ECL (PIERCE; Rockford, IL), following standard protocols.

### Transport assays

For uptake experiments, oocytes were washed four times with ND96 medium (pH 7.4) without pyruvate and gentamycin. After approximately 60 min of starvation, oocytes were preincubated in the indicated isotonic uptake medium [(96 mmol/L NaCl, 2.0 mmol/L KCl, 1.8 mmol/L CaCl_2_, 1.0 mmol/L MgCl_2_, and 5 mmol/L Hepes, pH 7.4) or (98 mmol/L KCl, 1.8 mmol/L CaCl_2_, 1.0 mmol/L MgCl_2_, and 5 mmol/L Hepes, pH 7.4) or Cl^‐^ free, uptake medium (100 mmol/L Na‐gluconate, k‐gluconate or, NMDG‐gluconate, 2 mmol/L k‐gluconate, 1 mmol/L Mg‐gluconate, 1 mmol/L Ca‐gluconate, 10 mmol/L HEPES, pH 7.4,) for about 30 min in the absence or presence of indicated concentration of drugs [tranilast (Nuon Therapeutics Inc. San Francisco, CA), benzbromarone (SIGMA), or probenecid (SIGMA)] and then incubated in the same medium containing [^14^C]urate/[^14^C]nicotinate (40 *μ*mol/L) in the absence or presence of indicated concentration of drugs or organic anions for 1 h. In other sets of experiment, control or indicated cRNA‐injected protein‐expressing oocytes were preinjected with 50 nL of water or unlabeled test organic anions [100 mmol/L pyrazine carboxylate (PZA), pH 7.4] using fine‐tipped micropipettes and then transferred to the isotonic medium (86 mmol/L NaCl, 10 mmol/L PZA, 2.0 mmol/L KCl, 1.8 mmol/L CaCl_2_, 1.0 mmol/L MgCl_2_ and 5 mmol/L Hepes, pH 7.4) containing 10 mmol/L PZA or indicated other organic anions and incubated for 2 h for recovery before the uptake assay. After 60 min of incubation in the indicated uptake medium containing [^14^C]urate/[^14^C]nicotinate (40 *μ*mol/L) at room temperature [25°C] in a horizontal shaker‐incubator, oocytes (15–20 in each group) were washed three times with the ice‐cold uptake medium to remove external radioisotope.

For [^14^C]‐urate efflux studies, oocytes expressing ABCG2 were preinjected with 50 nL of 1500 *μ*mol/L [^14^C]‐urate dissolved in efflux medium (ND96, pH 7.4). Preinjected oocytes were then incubated in ND96 medium for 1 h at 16°C for recovery. After incubation, the oocytes were washed in cold ND96 medium four times to remove any external adhering [^14^C]‐urate from the oocytes and then subjected to efflux for 1 h at room temperature (~25°C) in ND96 medium (pH 7.4) in the absence or presence of drug.

Radioisotope content of each individual oocyte was measured by scintillation counter after solubilization in 0.3 mL of 10% (v/v) SDS and addition of 2.5 mL of scintillation fluid. All uptake experiments included at least 15 oocytes in each experimental group, with multiple controls as appropriate; statistical significance for individual experiments was defined as two‐tailed *P* < 0.05, and results were reported as means ± S.E. All the uptake experiments shown were performed at least three times for confirmation; data for each figure are from a single representative experiment. Statistical analyses including linear regressions were done using SigmaPlot software.

## Results

### Tranilast inhibits urate transport mediated by URAT1

**Figure 1:**
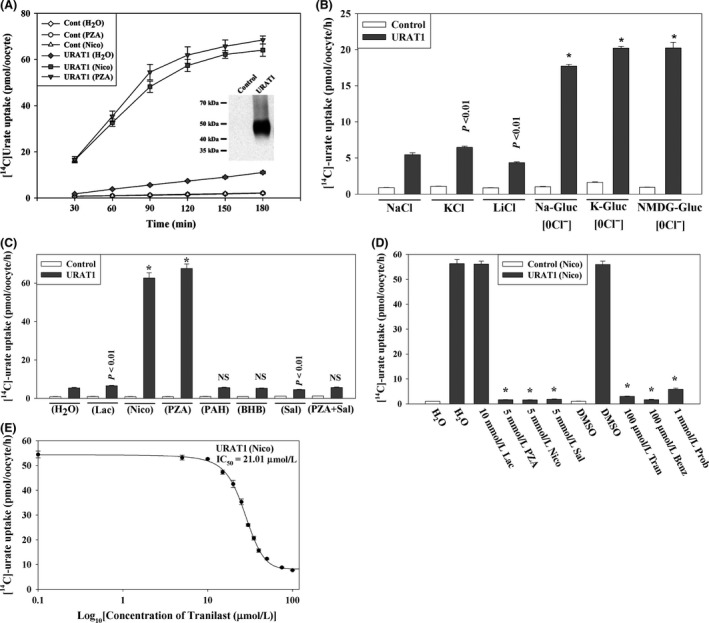
Tranilast inhibits urate transport mediated by the human urate transporter URAT1 expressed in *Xenopus laevis* oocytes. (A) Time course of [^14^C]‐urate uptake by control and URAT1‐expressing oocytes. [^14^C]‐urate uptake by URAT1‐expressing oocytes was trans‐stimulated by preinjecting with 50 nL of 100 mmol/L PZA or nicotinate (Nico) 2 h before [^14^C]‐urate uptake experiment. Urate uptakes in the control oocytes were identical, resulting in superimposed data. Inset, western blot analysis of URAT1 protein expressed in oocytes. (B) [^14^C]‐urate transport properties of URAT1: voltage sensitivity and Na^+^‐dependence was examined by replacing extracellular NaCl by KCl or LiCl. Chloride effects were also examined; for the 0 Cl^−^ bath, NaCl was replaced by Na‐gluconate (Na‐Gluc), KCl by K‐gluconate (K‐Gluc), MgCl_2_ by Mg‐gluconate, and CaCl_2_ by Ca‐gluconate. *P < 0.001 compared with NaCl (ND96). (C) Trans‐stimulatory effects of preloaded organic anions on [^14^C]‐urate uptake by URAT1‐expressing oocytes: [^14^C]‐urate uptake rate via URAT1 was measured in URAT1‐expressing oocytes preloaded with 50 nL of 100 mmol/L Lac, Nico, PZA, PAH, BHB or salicylate (Sal). *P < 0.001 compared with water‐injected control; NS, not significant. (D). Inhibition of [^14^C]urate by URAT1 in the presence of extracellular organic anions or uricosuric drugs, including tranilast: the uptake of [^14^C]‐urate (40 *μ*mol/L) by URAT1‐expressing oocytes in exchange of preloaded intracellular nicotinate was determined after 1 h in the absence or presence of inhibitors (uricosuric drugs or organic anions) that were added to the extracellular medium (pH 7.4) at the indicated concentrations. *P <* *0.001 compared with DMSO. Tran, Benz, Prob, DMSO. Data are mean ± S.E. with *n* = 12–15. (E) The 50% inhibitory concentration (IC_50_) curve of tranilast for [^14^C]‐urate uptake‐mediated by URAT1 in oocytes preloaded with nicotinate. PZA, pyrazine carboxylate; PAH, para‐aminohippurate; Tran, tranilast; Benz, benzbromarone; Prob, probenecid; DMSO, dimethylsulfoxide.

Multiple uricosuric drugs have been shown to inhibit human URAT1, one of three urate/anion exchangers at the apical membrane of human proximal tubule cells. URAT1 is thought to be the dominant urate‐anion exchanger in proximal tubular urate reabsorption, given that recessive loss‐of‐function mutations in the *SLC22A12* gene encoding URAT1 are associated with hypouricemia (Enomoto et al. 2002). To test the transport function of URAT1 in vitro, we preinjected URAT1 cRNA into *Xenopous* oocytes, examined URAT1 protein expression by western blotting, and then measured the [^14^C]‐urate uptake activity of the URAT1 in various isotonic uptake media at an extracellular [^14^C]‐urate concentration of 40 *μ*mol/L. We found that in isotonic ND96 medium (pH 7.4), the URAT1‐expressing oocytes showed about 5–6 fold higher [^14^C]‐urate uptake activity over the water‐injected control oocytes (Fig. 1A and B). When the Na^+^ in ND96 medium was substituted by K^+^ or Li^+^, the [^14^C]‐urate uptake activity of URAT1 was only slightly (*P* < 0.01) affected (Fig. 1B) which was in agreement with the previous observation (Enomoto et al. 2002) that urate transport via URAT1 is sodium‐independent. In chloride‐free medium (pH 7.4), URAT1 showed 17–21 fold higher [^14^C]‐urate uptake activity over the water‐injected control oocytes (Fig. 1B) suggesting either a capacity for urate/Cl^−^ exchange mechanism in URAT1 or unrelated cis‐inhibitory effects of extracellular Cl^−^. Antiuricosuric anions are thought to increase urate reabsorption by URAT1 through their trans‐stimulatory effects; therefore, we evaluated the trans‐stimulatory effect of preloaded intracellular pyrazinoate (PZA), nicotinate, lactate, p‐aminohippurate, *β*‐hydroxy butyrate, and salicylate on urate uptake via URAT1. When URAT1‐expressing oocytes were preloaded with nonlabeled PZA or nicotinate using a fine micropipette or by preincubating isotonic medium for 3–4 h, urate uptake activity was stimulated to 70–75 fold higher than PZA‐ or nicotinate‐preloaded control oocytes (Fig. 1C), which was consistent with previous observations (Enomoto et al. 2002). We did not however detect any significant trans‐stimulatory effect of preloaded intracellular lactate, para‐aminohippurate (PAH), and *β*‐hydroxybutyrate (BHB) on URAT1‐mediated urate uptake (Fig. 1C), perhaps due to metabolism of microinjected lactate and other anions. We also found that the URAT1‐mediated urate uptake was not cis‐inhibited by 10 mM extracellular organic anions such as lactate (Fig. 1D), pyruvate, BHB, PAH, *α*‐ketoglutarate (*α*‐KG), formate, oxalate, citrate, succinate, and maleate (data not shown), despite cis‐inhibition by 10 mmol/L extracellular nicotinate, PZA, and salicylate (Fig. 1D).

We then proceeded to examine the effect of varying concentrations of tranilast on URAT1‐mediated [^14^C]‐urate uptake. We found that tranilast inhibited basal URAT1‐mediated [^14^C]‐urate uptake, that is, uptake in the absence of PZA or nicotinate preloading, in a dose‐dependent manner with a 50% inhibitory concentration (IC_50_) of ~20 *μ*mol/L (data not shown). We also found that tranilast very efficiently inhibited URAT1‐mediated [^14^C]‐urate/PZA or [^14^C]‐urate/nicotinate exchange (Fig. 1D) in a dose‐dependent manner with an IC_50_ of **˜**21 *μ*mol/L (Fig. 1E). In parallel, we found that both benzbromarone (100 *μ*mol/L) and probenecid (1 mmol/L) also efficiently inhibited URAT1‐mediated [^14^C]‐urate/PZA or [^14^C]‐urate/nicotinate exchange (Fig. 1D) with an IC_50_ of benzbromarone ~0.45 *μ*mol/L for URAT1‐mediated [^14^C]‐urate uptake or [^14^C]‐urate/PZA exchange (data not shown).

### Tranilast inhibits urate transport mediated by GLUT9a

**Figure 2:**
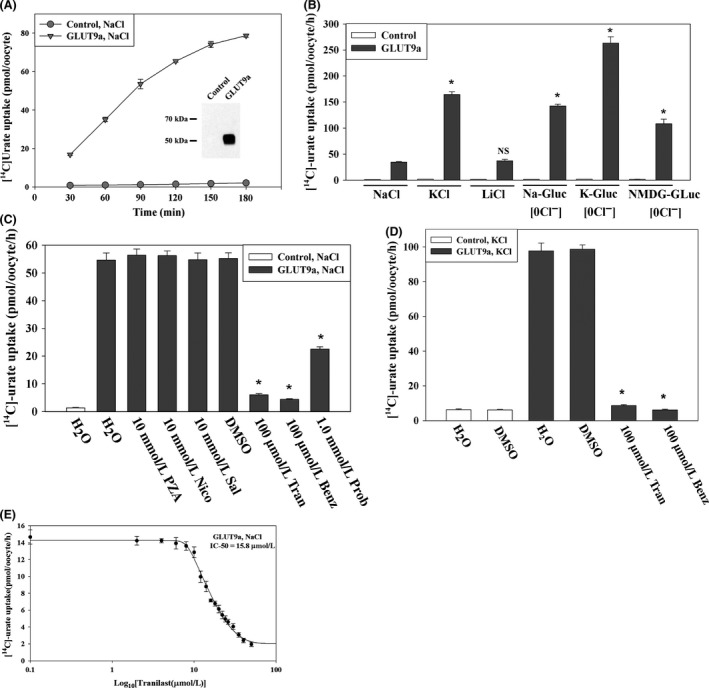
Tranilast inhibits urate transport mediated by the human urate transporter GLUT9a. (A) Time course of [^14^C]‐urate uptake by control and GLUT9a‐expressing oocytes. Inset, western blot analysis of GLUT9a protein expressed in oocytes. (B) [^14^C]urate transport properties of GLUT9a: voltage sensitivity and Na^+^‐dependence was examined by replacing extracellular NaCl by KCl or LiCl. We also assessed [^14^C]‐urate uptake by GLUT9a in the absence of extracellular Cl^−^ ion (0 Cl^−^); in 0 Cl^−^ bath, NaCl was replaced by Na‐gluconate (Na‐Gluc), KCl by k‐gluconate (K‐Gluc), MgCl_2_ by Mg‐gluconate, and CaCl_2_ by Ca‐gluconate. Asterisk, *P *<* *0.001 compared with NaCl (ND96); NS, not significant. (C) Inhibition of [^14^C]‐urate uptake by GLUT9a in the presence of antiuricosuric or uricosuric drugs: The uptake of [^14^C]‐urate (40 *μ*mol/L) by GLUT9a‐expressing oocytes was determined in complete absence of Na^+^ (NaCl of ND96 medium was replaced by KCl) after 1 h in the absence or presence of inhibitors (uricosuric drugs) which were added to the extracellular medium (pH 7.4) at the indicated concentrations. *P <* *0.001 compared with DMSO. (D) Inhibition of [^14^C]‐urate uptake by GLUT9a in depolarized cells. The uptake of [^14^C]‐urate (40 *μ*mol/L) by GLUT9a‐expressing oocytes was determined in the complete absence of Na^+^ (NaCl of ND96 medium was replaced by KCl) after 1 h in the absence or presence of inhibitors (uricosuric drugs) at the indicated concentrations. *P <* *0.001 compared with DMSO. Tran, Benz Prob, Sal, DMSO. Data are mean ± S.E. with *n* = 12–15. Data are mean ± S.E. with *n* = 12–15. (D) The 50% inhibitory concentration (IC_50_) curve of tranilast for [^14^C]‐urate uptake via GLUT9a. Tran, tranilast; Benz, benzbromarone; Prob, probenecid; Sal, salicylate; DMSO, dimethylsulfoxide.

Human GLUT9‐mediated urate transport has been shown to be electrogenic and affected by changes in membrane potential (Anzai et al. 2008; Bibert et al. 2009; Witkowska et al. 2012). GLUT9 has two isoforms with divergent N‐terminal cytoplasmic domains, generated by transcriptional initiation at alternative promoters; these isoforms have equivalent urate transport activity when expressed in *Xenopus laevis* oocytes (Anzai et al. 2008). We sought to investigate the effect of tranilast on GLUT9a‐mediated [^14^C]‐urate uptake in *Xenopus laevis* oocytes. We found that the [^14^C]‐urate uptake activity of GLUT9a‐expressing oocytes was 37–39 fold higher than that of water‐injected control oocytes (Fig. 2A and B) in isotonic ND96 uptake medium at an extracellular [^14^C]‐urate concentration of 40 *μ*mol/l [^14^C]‐urate. The urate uptake activity of GLUT9a in depolarized oocytes (Fig. 2B), induced by the replacement of extracellular Na^+^ by K^+^, was found to be 140–150 fold higher than control oocytes. The complete removal of Cl^−^ ion from the extracellular medium also increased the urate uptake activity of GLUT9a, to130–140 fold over control (Fig. 2B). This urate uptake was efficiently inhibited by both tranilast (100 *μ*mol/L) and benzbromarone (100 *μ*mol/L) but only about 60% inhibited by probenecid (1.0 mmol/L) (Fig. 2 C and D); PZA, nicotinate, and salicylate had no effect on GLUT9a activity (Fig. 2C). Tranilast inhibited GLUT9a‐mediated [^14^C]‐urate uptake in a dose‐dependent manner with an IC_50_ of ~15.6 *μ*mol/L (Fig. 2E); for benzbromarone, the IC_50_ was ~14.2 *μ*mol/L (data not shown). The IC_50_ of tranilast for GLUT9a remained unchanged by membrane depolarization, that is, the drug was equally effective under both membrane‐polarized and depolarized conditions (data not shown).

### Tranilast inhibits urate transport mediated by OAT4

**Figure 3:**
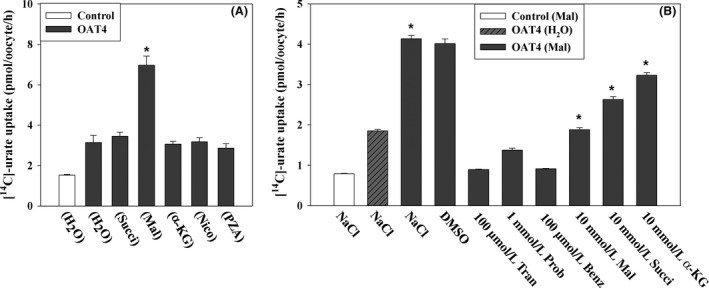
Tranilast inhibits urate transport mediated by the human urate/dicarboxylate exchanger OAT4. (A) The uptake of [^14^C]‐urate by OAT4‐expressing oocytes was performed in ND96 medium (pH 7.4) at ~25°C for 1 h. Na^+^‐dependence was examined by replacing extracellular NaCl by KCl. The [^14^C]‐urate uptake by OAT4‐expressing oocytes was found to be significantly trans‐stimulated when OAT4‐expressing oocytes were preinjected with 50 nL of 100 mmol/L maleate (Mal); preinjection with 50 nL of 100 mmol/L succinate (Succi), *α*‐ketogluterate (*α*‐KG), nicotinate (Nico), or PZA had no significant effects. Asterisk, *P* < 0.001 compared with water‐injected control. (B) The inhibition of [^14^C]‐urate uptake by OAT4 was examined in the presence of extracellular organic anions (for cis‐inhibition) or uricosuric drugs at the indicated concentrations. Asterisk, *P *<* *0.001 compared with DMSO/NaCl(ND96). Tran, Benz, Prob, DMSO. Data are mean ± S.E. with *n* = 12–15. PZA, pyrazine carboxylate; Tran, Tranilast; Benz, Benzbromarone; Prob, probenecid; DMSO, dimethylsulfoxide; Lac, lactate; Nico, nicotinate.

Human OAT4 reportedly functions as an apical urate/dicarboxylate exchanger at the apical membrane of renal proximal tubule cells (Hagos et al. 2007). We sought to investigate whether tranilast inhibits [^14^C]‐urate uptake activity of OAT4 in *Xenopus laevis* oocytes that express OAT4 protein. We found that the OAT4‐mediated [^14^C]‐urate uptake was approximately twofold higher than that of water‐injected control oocytes (Fig. 3A) in ND96 medium. Preloading with intracellular maleate had a modest trans‐stimulatory effect on OAT4‐mediated urate uptake (Fig. 3A) compared to other preloaded organic anions. We also found that the OAT4‐mediated trans‐stimulation of urate uptake by maleate was efficiently cis‐inhibited by 10 mM extracellular maleate with much less cis‐inhibition by 10 mmol/L succinate or *α*‐KG (Fig. 3B). OAT4‐mediated [^14^C]‐urate uptake was very efficiently inhibited by tranilast (100 *μ*mol/L), benzbromarone (100 *μ*mol/L), but only 80% inhibited by probenecid (1 mmol/L) (Fig. 3B). The IC_50_ of tranilast for [^14^C]‐urate uptake by OAT4 was found to be ~22 *μ*mol/L (data not shown).

### Tranilast inhibits urate transport mediated by OAT10

**Figure 4:**
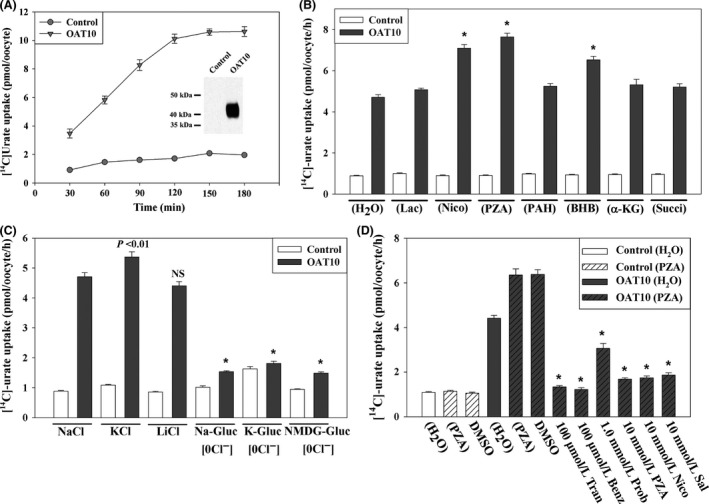
Tranilast inhibits urate transport mediated by the human urate transporter OAT10. (A) Time course of [^14^C]‐urate uptake by control and URAT1‐expressing oocytes. [14C]‐urate uptake by OAT10‐expressing oocytes was trans‐stimulated by preinjecting with 50 nL of 100 mmol/L PZA 2 h. Inset, western blot analysis of OAT10 protein expressed in oocytes. (B) Trans‐stimulatory effects of preloaded organic anions on [^14^C]‐urate uptake by OAT10‐expressing oocytes: [^14^C]‐urate uptake rate mediated by OAT10 was measured in OAT10‐expressing oocytes preloaded with 50 nL of 100 mmol/L Lac, Nico, PZA, PAH, BHB,* α*‐ketoglutarate (*α*‐KG) or succinate (Succi). *P < 0.001 compared with water‐injected control. (C) [^14^C]‐urate transport properties of OAT10: voltage sensitivity and Na+‐dependence was examined by replacing extracellular NaCl by KCl or LiCl. Existence of urate/Cl^−^ exchange mechanism or Cl^−^ dependence or cis‐inhibitory effects of extracellular Cl^−^ on was verified by [^14^C]‐urate uptake via OAT10 in the absence of extracellular Cl^−^ ion (0 Cl^−^). In 0 Cl^−^ bath, NaCl was replaced by Na‐gluconate (Na‐Gluc), KCl by k‐gluconate (K‐Gluc), MgCl2 by Mg‐gluconate, and CaCl_2_ by Ca‐gluconate. Asterisk, *P* < 0.001 compared with NaCl (ND96); NS, not significant. (D) Inhibition of [^14^C]‐urate by OAT10 in the presence of extracellular organic anions or uricosuric drugs: The uptake of [^14^C]‐urate (40 *μ*mol/L) by OAT10‐expressing oocytes in exchange of preloaded intracellular PZA was determined after 1 h in the absence or presence of inhibitors (uricosuric drugs) that were added to the extracellular medium at the indicated concentrations. *P < 0.001 compared with NaCl (ND96). Tran, Benz, Prob, DMSO. Data are mean ± S.E. with *n* = 12–15. (E) The 50% inhibitory concentration (IC_50_) curve of tranilast for [^14^C]‐urate uptake via OAT10 in oocytes preloaded with PZA. PZA, pyrazine carboxylate; PAH, para‐aminohippurate; Lac, lactate; Nico, nicotinate; PZA, pyrazine carboxylate; Tran, tranilast; Benz, benzbromarone; Prob, probenecid; DMSO, dimethylsulfoxide.

The hORCTL3/OAT10 (human organic cation transporter like 3/organic anion transporter‐10, encoded by *SLC22A13*) has been characterized as a urate transporter and high‐affinity nicotinate exchanger (Bahn et al. 2008); it functions in apical urate reabsorption in the proximal tubule. We sought to investigate whether tranilast inhibits [^14^C]‐urate uptake activity of OAT10 in *Xenopus laevis* oocytes that express OAT10 protein. We found that the OAT10‐mediated [^14^C]‐urate uptake was ~4–5 fold higher than that of water‐injected control oocytes (Fig. 4A–C) in ND96 medium. Preloading with the intracellular organic anions, PZA, nicotinate or BHB, had a modest trans‐stimulatory effect on OAT10‐mediated urate uptake (Fig. 4B). In chloride‐free medium (pH 7.4), OAT10 showed negligible [^14^C]‐urate uptake activity (Fig. 4C) indicating OAT10‐mediated urate transport is dependent on extracellular Cl^−^, which is in marked contrast with the behavior of URAT1. OAT10‐mediated [^14^C]‐urate uptake was very efficiently inhibited by both tranilast (100 *μ*mol/L) and benzbromarone (100 *μ*mol/L), however, probenecid (1.0 mmol/L) inhibited about 65% of the urate transport activity of OAT10 (Fig. 4D). The IC_50_ of tranilast for [^14^C]‐urate uptake by OAT10 was found to be ~31 *μ*mol/L, and for benzbromarone, it was ~20 *μ*mol/L (data not shown).

### Tranilast inhibits urate transport via URAT1 and GLUT9a in a reversible, noncompetitive (mixed) manner

To determine the reversibility of urate transport inhibition, oocytes expressing URAT1 or GLUT9a were preincubated with 100 *μ*mol/L tranilast and then subjected to [^14^C]‐urate uptake for 1 h after washing and removal of the inhibitor. We found almost no inhibition of urate transport mediated by URAT1 (Fig. S1) or GLUT9a (Fig. S2) after removal of tranilast from the uptake medium. When similar experiments were performed in parallel for benzbromarone, the urate uptake was substantially inhibited even in the absence of additional benzbromarone (Figs. 1, 2). These results indicate that tranilast reversibly inhibited URAT1‐ or GLUT9a‐mediated urate transport, whereas inhibition of URAT1‐ and GLUT9a‐mediated urate transport by benzbromarone was not immediately reversible.

**Figure 5:**
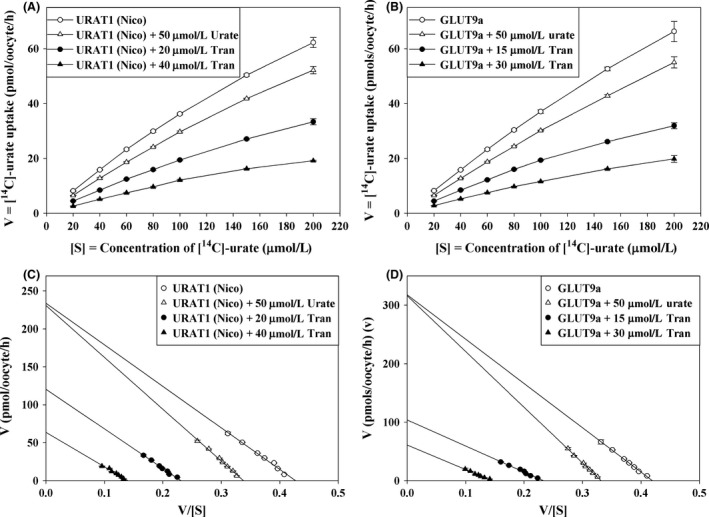
Tranilast inhibits urate transport activity of URAT1 and GLUT9a in a noncompetitive (mixed) manner. (A) The kinetic curve of URAT1‐mediated [^14^C]‐urate uptake in the absence or presence of inhibitor (cold urate or tranilast). (B) The kinetic curve of GLUT9a‐mediated [^14^C]‐urate uptake in the absence or presence of inhibitor (cold urate or tranilast). (C) Eadie–Hofstee plot of URAT1‐mediated [^14^C]‐urate uptake in the absence or presence of inhibitor. (D) Eadie–Hofstee plot of GLUT9a‐mediated [^14^C]‐urate uptake in the absence or presence of inhibitor. V, the [^14^C]‐urate uptake rate in pmol/oocyte/h; V/S, [^14^C]‐urate uptake rate per concentration (*μ*mol/L) of [^14^C]‐urate; open circle, in the absence of inhibitor; open circle, in the absence of inhibitor; open triangle, in the presence 50 *μ*mol/L cold urate; closed circle 15 or 20 *μ*mol/L tranilast; closed triangle 30 or 40 *μ*mol/L tranilast. Tran, Benz, Prob, SalDMSO, W, oocytes were washed out of drugs with uptake medium. Data are mean ± S.E. with *n* = 12–15. Tran, Tranilast; Benz, Benzbromarone; Prob, probenecid; Sal, salicylate; DMSO, dimethylsulfoxide; W, oocytes were washed out of drugs with uptake medium.

To further characterize the mechanism of inhibition of urate transport caused by tranilast, we examined the kinetics of both URAT1 and GLUT9a‐mediated [^14^C]‐urate uptake for 1 h at room temperature [25°C], using increasing concentrations of [^14^C]‐urate in ND96 medium (pH 7.4) containing no inhibitor, 50 *μ*mol/L cold urate, or 15–40 *μ*mol/L tranilast (Fig. 5A and B) and then analyzing the mode of inhibition after Eadie–Hofstee linearizations (Fig. 5C and D). The results shown in Figure 5C demonstrate that the inhibition of URAT1‐mediated urate transport by tranilast closely resembles a noncompetitive inhibition (mixed) model since the *V*_max_ was decreased from 233.4 to 120.1 pmoles/oocyte/h and the apparent *K*_m_ (*K*_m_^app^) was decreased from 548 *μ*mol/L to 522 *μ*mol/L by 20 *μ*mol/L of tranilast. In the presence of 40 *μ*mol/L of tranilast, the *V*_max_ was decreased from 233.4 to 63.5 pmoles/oocyte/h and the apparent *K*_m_ (*K*_m_^app^) was decreased from 548 *μ*mol/L to 460 *μ*mol/L. The inhibition constant (*K*_i_) of tranilast for URAT1‐mediated urate uptake was determined to be 21.33 ± 2.2 *μ*mol/L. As expected for a noncompetitive mechanism (Cheng and Prusoff 1973), the inhibition constant is equivalent to the measured IC_50_ for URAT1.

For GLUT9a, (Fig. 5D) the *V*_max_ was decreased from 317.7 to 108.4 pmoles/oocyte/h and the apparent *K*_m_ (*K*_m_^app^) was decreased from 757.5 *μ*mol/L to 452.6 *μ*mol/L by 15 *μ*mol/L of tranilast. In the presence of 30 *μ*mol/L of tranilast, the Vmax was decreased from 317.7 to 61 pmoles/oocyte/h and the apparent *K*_m_ (*K*_m_^app^) was decreased from 757.51 *μ*mol/L to 420 *μ*mol/L. Therefore, the results shown in Figure 5D demonstrate that the inhibition of GLUT9a‐mediated urate transport by tranilast also closely resembles a noncompetitive (mixed) inhibition model. The inhibition constant (*K*_i_) of tranilast for GLUT9a‐mediated urate uptake was determined to be 17.13 ± 1.3 *μ*mol/L. As expected for a noncompetitive mechanism (Cheng and Prusoff 1973), the inhibition constant is equivalent to the measured IC_50_ for GLUT9a.

### URAT1‐mediated nicotinate transport is resistant to tranilast

**Figure 6:**
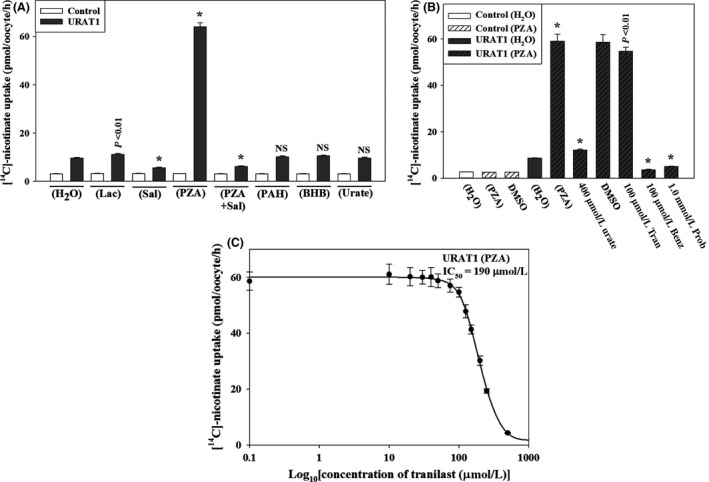
Functional characterization of human URAT1 as a nicotinate transporter; effects of uricosuric drugs. (A) Trans‐stimulatory effects of preloaded organic anions on [^14^C]‐nicotinate uptake by URAT1‐expressing oocytes: [^14^C]‐nicotinate transport rate via URAT1 was measured in URAT1‐expressing oocytes preloaded with 50 nL of 100 mmol/L Lac, PZA, PAH, BHB, or urate. *P <* *0.001 compared with water‐injected control; NS, not significant. (B) Inhibition of [^14^C]‐nicotinate by URAT1 in the presence of extracellular urate or uricosuric drugs: The uptake of [^14^C]‐nicotinate (40 *μ*mol/L) by URAT1‐expressing oocytes in exchange of preloaded intracellular PZA was determined after 1 h in the absence or presence of inhibitors (uricosuric drugs) that were added to the extracellular medium (pH 7.4) at the indicated concentrations. *P <* *0.001 compared with DMSO/NaCl(ND96). Tran, Benz, Prob, DMSO. (C) The 50% inhibitory concentration (IC_50_) curve of tranilast for [^14^C]‐nicotinate uptake by URAT1‐expressing oocytes preinjected with 50 nL of 100 mmol/L PZA 2 h before [^14^C]‐nicotinate uptake experiment. Data are mean ± S.E. with *n* = 12–15. BHB*, β*‐hydroxy butyrate; PAH, para‐aminohippurate; Lac, lactate, PZA, pyrazine carboxylate; PAHe , para‐aminohippurate; Tran, Tranilast; Benz, Benzbromarone.

Since organic anions such as nicotinate and pyrazine carboxylate (PZA) were found to trans‐stimulate as well as cis‐inhibit URAT1‐mediated urate uptake (Fig. 1 A, C and D), we sought to determine whether extracellular [^14^C]‐nicotinate is transported by URAT1 and whether this activity is sensitive to tranilast. We found that the URAT1‐expressing oocytes showed ~2 fold higher [^14^C]‐nicotinate uptake activity over water‐injected control oocytes in ND96 medium at an extracellular [^14^C]‐nicotinate concentration of 40 *μ*mol/L; this activity was time‐dependent (Fig. S3), Na^+^‐independent, and inhibited by extracellular Cl^−^ (Fig.S4). Preloading with PZA had a significant trans‐stimulatory effect (about 19–20 fold higher than the water‐injected control oocytes) on URAT1‐mediated [^14^C]‐nicotinate uptake (Fig. 6A) compared to other preloaded organic anions. We then examined the effect of varying concentrations of tranilast on URAT1‐mediated [^14^C]‐nicotinate/PZA exchange. Surprisingly, we found that URAT1‐mediated [^14^C]‐nicotinate uptake was significantly resistant to tranilast (Fig. 6B) with an IC_50_ of ~190 *μ*mol/L (Fig. 6C), whereas [^14^C]‐nicotinate uptake remained very sensitive to benzbromarone (IC_50_ ~0.42 micromol/L, i.e. using the Greek for micro as in all the other IC_50_s) and probenecid (data not shown). The URAT1‐mediated [^14^C]‐nicotinate uptake was also cis‐inhibited by the presence of extracellular urate, PZA, or salicylate (data not shown).

### SMCT1‐ and SMCT2‐mediated nicotinate transport is resistant to tranilast

**Figure 7:**
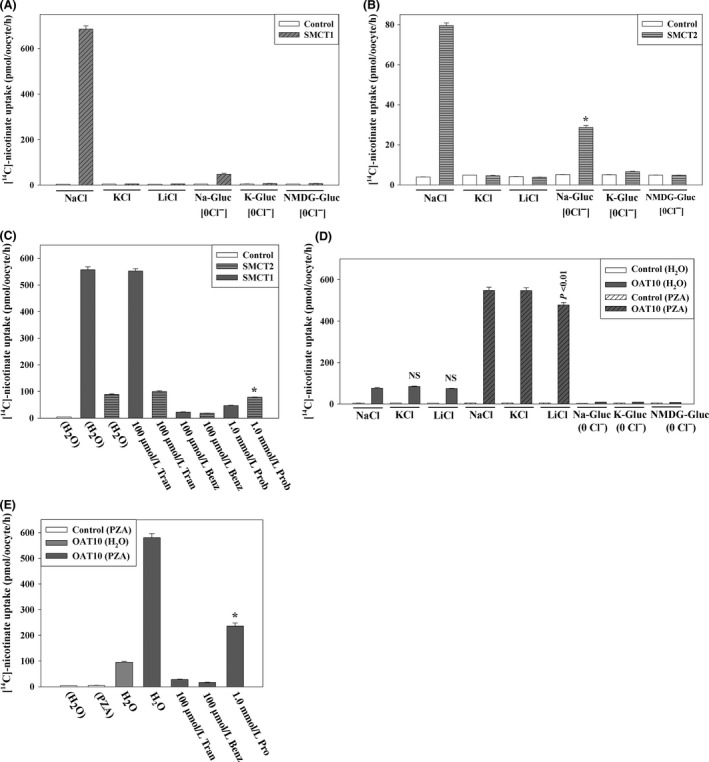
Functional and pharmacological characterization of human nicotinate transporters, hSMCT1, SMCT2, and OAT10. The uptake of [^14^C]‐nicotinate (40 *μ*mol/L) by SMCT1‐, SMCT2‐ or OAT10‐expressing oocytes was performed in ND96 medium (pH 7.4) at ~25°C. [^14^C]‐nicotinate transport properties of nicotinate transporters: voltage sensitivity and Na^+^‐dependence was examined by replacing extracellular NaCl by KCl or LiCl. Chloride ion‐dependence was also assessed; in 0 Cl^−^ bath, NaCl was replaced by Na‐gluconate (Na‐Gluc), KCl by K‐gluconate (K‐Gluc), MgCl_2_ by Mg‐gluconate, and CaCl_2_ by Ca‐gluconate. (A) [^14^C]‐nicotinate transport properties of human SMCT1 (B) [^14^C]nicotinate transport properties of human SMCT2. *P <* *0.001 compared with NaCl (ND96). (C) Inhibition of [^14^C]‐nicotinate uptake via SMCT1, and SMCT2 in the presence of uricosuric drugs added into the extracellular medium (pH 7.4) at the indicated concentrations. *P *<* *0.01 compared with NaCl (ND96); NS, not significant. (D) [^14^C]‐nicotinate transport properties of human OAT10 in oocytes preloaded with PZA. *P <* *0.001 compared with uptake in the absence of inhibitor; NS, not significant. (E) Inhibition of [^14^C]‐nicotinate uptake via OAT10 (preloaded with PZA) in the presence of uricosuric drugs added into the extracellular medium (pH 7.4) at the indicated concentrations. *P *<* *0.01 compared with NaCl (ND96); NS, not significant. Tran, Benz, Prob, DMSO. Data are mean ± S.E. with *n* = 12–15. PZA, pyrazine carboxylate; Tran, Tranilast; Benz, Benzbromarone; Prob, probenecid; DMSO, dimethylsulfoxide.

SMCT1 (encoded by *SLC5A8*) and SMCT2 (encoded by *SLC5A12*) are sodium‐coupled renal monocarboxylate transporters (SMCTs), the former high‐affinity and the latter low‐affinity (Coady et al. 2004; Srinivas et al. 2005). SMCT1 and SMCT2 transport a variety of monocarboxylates, including nicotinate, lactate, and pyruvate, in a Na^+^‐dependent manner (Coady et al. 2004; Srinivas et al. 2005). The transport of urate via URAT1 is known to be driven by the intracellular nicotinate or PZA concentration, such that by loading cells with these substrates, SMCT1 and SMCT2 collaborate in the proximal tubule with URAT1 and OAT10 in apical urate reabsorption (Mandal and Mount 2015). We sought to investigate the effect of tranilast on SMCT1‐ and SMCT2‐mediated [^14^C]‐nicotinate uptake in oocytes. We first examined the [^14^C]‐nicotinate uptake activity of SMCT1 and SMCT2 expressed in *Xenopous* oocytes 48 h after injection of the respective cRNA. We found that SMCT1‐mediated [^14^C]‐nicotinate uptake activity was Na^+^‐, Ca^2+^‐, and Cl^−^ ion‐dependent and 150–160 fold higher than the water‐injected control oocytes in ND96 medium (Fig. 7A). The SMCT2‐mediated [^14^C]‐nicotinate uptake activity was primarily Na^+^ ion‐dependent and 17–18 fold higher than the water‐injected control oocytes in ND96 medium (Fig. 7B). Both SMCT1 and SMCT2‐mediated [^14^C]‐nicotinate uptake were found to be very resistant to tranilast (IC_50_~1.1 mmol/L for SMCT1 and IC_50_~1.0 mmol/L for SMCT2) (data not shown) but sensitive to benzbromarone (IC_50_~49.6 *μ*mol/L for SMTC1 and ~61.3 μmol/L for SMCT2) (data not shown). SMCT1 was significantly inhibited by 1.0 mmol/L probenecid, whereas SMCT2 remained almost unaffected (Fig. 7C).

### OAT10‐mediated nicotinate transport is sensitive to tranilast

Since OAT10 is a high‐affinity nicotinate exchanger (Bahn et al. 2008), we sought to investigate the effect of tranilast on OAT10‐mediated [^14^C]‐nicotinate uptake through its expression in *Xenopus laevis* oocytes. We first examined the [^14^C]‐nicotinate uptake activity of OAT10 expressed in *Xenopous* oocytes 48 h after preinjection of the respective cRNA. We found that the human OAT10‐mediated [^14^C]‐nicotinate uptake was 17–18 fold higher than the water‐injected control oocytes (Fig. 7D) in ND96 medium. In chloride‐free medium (pH 7.4), OAT10 showed very little [^14^C]‐nicotinate uptake activity over the water‐injected control oocytes (Fig. 7D) indicating that, in comparison to URAT1, OAT10‐mediated [^14^C]‐nicotinate uptake activity is Cl^−^ ion‐dependent.

The [^14^C]‐nicotinate uptake activity of OAT10 was found to be inhibited by both tranilast (100 *μ*mol/L) and benzbromarone (100 *μ*mol/L)) (Fig. 7E). Tranilast was found to inhibit OAT10‐mediated [^14^C]‐nicotinate uptake with an IC_50_ of ~42**** *μ*mol/L (data not shown). When OAT10‐expressing oocytes were preloaded with nonlabeled PZA, the [^14^C]‐nicotinate uptake activity increased to 118–120 fold higher than PZA‐preloaded control oocytes (Fig. 7E and F) in ND96 medium. Tranilast also inhibited OAT10‐mediated enhanced [^14^C]‐nicotinate/PZA exchange with the IC_50_ of **˜**43.1 μmol/L (Fig. S5). In parallel, benzbromarone was found to inhibit OAT10‐mediated [^14^C]‐nicotinate/PZA exchange with the IC_50_ of ~22.8 *μ*mol/L (data not shown). Therefore, the OAT10‐mediated nicotinate transport is more sensitive to tranilast which is in marked contrast with that of SMCT1, SMCT2, and URAT1 which are resistant to tranilast (IC_50_ > 185 *μ*mol/L).

### Tranilast inhibits urate transport mediated by NPT1

**Figure 8:**
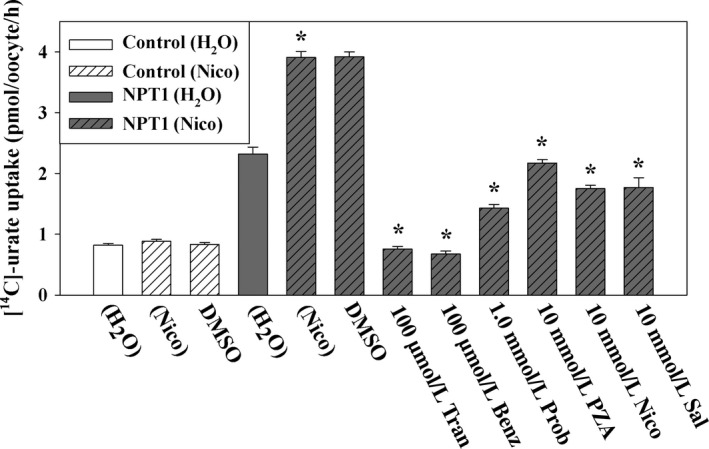
Tranilast inhibits urate transport mediated by the human urate transporter NPT1. The uptake of [^14^C]‐urate by NPT1‐expressing oocytes was performed in ND96 medium (pH 7.4) at ~25°C for 1 h. The [^14^C]‐urate uptake by NPT1‐expressing oocytes was found little trans‐stimulated when NPT1‐expressing oocytes were preinjected with 50 nL of 100 mmol/L nicotinate (Nico) 2 h before [^14^C]‐urate uptake experiment. The [^14^C]‐urate uptake by NPT1 was found substantially inhibited in the presence of extracellular organic anions or uricosuric drugs. Organic anions or uricosuric drugs were added to the extracellular medium (pH 7.4) at the indicated concentrations. *P <* *0.001 compared with uptake in the presence of DMSO or in the absence of inhibitor. Tran, Benz, Prob, DMSO, PZA, Sal. Data are mean ± S.E. with *n* = 12–15. PZA, pyrazine carboxylate; Tran, Tranilast; Benz, Benzbromarone; Prob, probenecid; DMSO, dimethylsulfoxide.

The results of our previous experiments have clearly established that tranilast inhibits almost all the known urate transporters in the urate reabsorptive pathway. Human NPT1 (Na^+^‐phosphate transporter‐1, encoded by *SLC17A1*) has been reported to transport organic anions such as urate, PAH, aspirin, and salicylate in a voltage‐driven and Cl^−^‐dependent manner (Iharada et al. 2010). NPT1 is expressed in the apical membrane of renal tubular cells and mediates urate secretion. We investigated whether tranilast inhibits [^14^C]‐urate uptake activity of NPT1 in *Xenopus laevis* oocytes. NPT1‐mediated [^14^C]‐urate uptake was found to be effectively inhibited by tranilast (100 *μ*mol/L) and benzbromarone (100 *μ*mol/L) but only partially inhibited by probenecid (1.0 mmol/L) (Fig. 8). The IC_50_ of tranilast for [^14^C]‐urate uptake by NPT1 was ~18.9 *μ*mol/L and for benzbromarone it was ~17.1 *μ*mol/L (data not shown).

### ABCG2‐mediated urate efflux is not affected by tranilast

**Figure 9:**
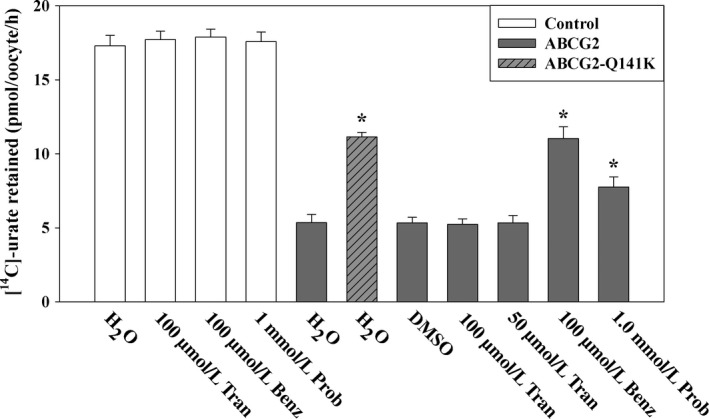
Urate efflux mediated by the human urate transporter ABCG2 is unaffected by tranilast. Control or ABCG2‐expressing oocytes were preinjected with 50 nL of 500 *μ*mol/L ^14^C]‐urate [dissolved in efflux medium (ND96, pH 7.4)], incubated in ND96 medium for 1 h at 16°C for recovery. Oocytes were washed in cold efflux medium four times and then subjected to efflux for 1 h at room temperature (~25°C) in ND96 medium (pH 7.4) in the absence or presence of drug. The amount of [^14^C]‐urate retained in each oocyte after 60 min of efflux is shown here. *P <* *0.001 compared with urate efflux in the absence of any inhibitor. Tran, Benz, Prob, DMSO. All data are mean ± S.E.M. with *n* = 12–15. PZA, pyrazine carboxylate; Tran, Tranilast; Benz, Benzbromarone; Prob, probenecid; DMSO, dimethylsulfoxide. Prob, probenecid; DMSO, dimethylsulfoxide.

The ATP‐binding cassette, subfamily G, member 2 (ABCG2) protein has been recently characterized as a high‐capacity urate secretion transporter (Woodward et al. 2009; Matsuo et al. 2012) expressed on the apical membrane of human renal proximal tubular cells (Huls et al. 2008), in addition to enterocytes and hepatocytes. We investigated the effect of tranilast (added in the efflux assay medium) on the [^14^C]‐urate efflux activity of ABCG2 expressed in *Xenopus laevis* oocytes. For [^14^C]‐urate efflux studies, control and ABCG2‐expressing oocytes were preinjected with 50 nL of 500 *μ*mol/L [^14^C]‐urate and then incubated in the in ND96 medium (pH 7.4) for 1 h for recovery before efflux experiment. Preinjected oocytes were washed to remove any residual extracellular [^14^C]‐urate and then subjected to [^14^C]‐urate efflux for 1 h at room temperature (~25°C) in the absence or presence of tranilast. The results of this urate efflux experiment show that human ABCG2‐mediated urate efflux remained unaffected by tranilast at even 100 *μ*mol/L concentration (Fig. 9), whereas benzbromarone (100 *μ*mol/L) inhibited ~48% and probenecid (1.0 mmol/L) inhibited ~20% of urate transport activity of ABCG2. The mutation (Q141K) in ABCG2 causing hyperuricemia and gout (Woodward et al. 2009) showed ~50% of the urate transport activity of wild‐type ABCG2 (Fig. 9).

### Tranilast inhibits urate transport mediated by OAT3 and OAT1

**Figure 10:**
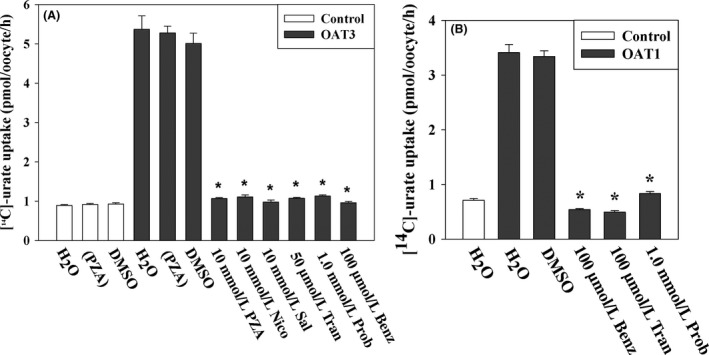
Tranilast inhibits urate transport mediated by urate transporter OAT3 and OAT1. The uptake of [^14^C]‐urate (40 *μ*mol/L) was performed in ND96 medium (pH 7.4) at ~25°C for 1 h. (A) The [^14^C]‐urate uptake by mouse OAT3 in oocytes remained unaffected if oocytes were preinjected with 50 nL of 100 mmol/L PZA but cis‐inhibited by 10 mmol/L PZA, nicotinate (Nico), or salicylate (Sal). The [^14^C]‐urate uptake by OAT3‐expressing oocytes was effectively inhibited by 50 *μ*mol/L tranilast (Tran), 100 *μ*mol/L benzbromarone (Benz) or 1.0 mmol/L probenecid (Prob). (B) The [^14^C]‐urate uptake by human OAT1 in oocytes was effectively inhibited by 100 *μ*mol/L tranilast (Tran), 100 *μ*mol/L benzbromarone (Benz) or 1.0 mmol/L probenecid (Prob). Data are mean ± S.E. with *n* = 12–15. *P < 0.001 compared with uptake in the presence of DMSO or in the absence of inhibitor. PZA, pyrazine carboxylate; Tran, Tranilast; Benz, Benzbromarone; Prob, probenecid; DMSO, dimethylsulfoxide.

Basolateral entry of urate into proximal tubule cells via OAT1 and OAT3 mediates the first step of urate secretion by this nephron segment (Eraly et al. 2008). We investigated the effect of tranilast on [^14^C]‐urate uptake activity of OAT3 in *Xenopous laevis* oocytes. We found that the OAT3‐mediated [^14^C]‐urate uptake was ~5 fold higher than the water‐injected control oocytes (Fig. 10A) in ND96 medium. Preloaded intracellular organic anion such as PZA and nicotinate did not have any effect on its [^14^C]‐urate uptake (data not shown). The OAT3‐mediated urate uptake was however almost completely cis‐inhibited by extracellular 10 mmol/L PZA, nicotinate or salicylate (Fig. 10A). OAT3‐mediated urate uptake was also effectively inhibited by 50 *μ*mol/L tranilast, with an IC_50_ of ~15.0 *μ*mol/L (data not shown), 100 *μ*mol/L benzbromarone, and 1.0 mmol/L probenecid (Fig. 10A). We also found that OAT1‐mediated urate uptake was ~5 fold higher than the water‐injected control oocytes (Fig. 10B) in ND96 medium and it was effectively inhibited by tranilast (100 *μ*mol/L), benzbromarone (100 *μ*mol/L) and probenecid (1.0 mmol/L) (Fig. 10B).

## Discussion

In humans, the reabsorption of filtered urate by the renal proximal tubule predominates over proximal tubular urate secretion, such that even modest inhibition of reabsorptive urate transport can cause uricosuria (Roch‐Ramel and Guisan 1999). Tranilast, an anti‐inflammatory drug, causes a potent reduction in SUA in human subjects, at least partially due to uricosuria (Sundy and Kitt 2010), hence we have investigated the effect of this drug on reabsorptive urate transporters. Here, we report that tranilast inhibits urate transport mediated by all the major reabsorptive urate transporters, as expressed in *Xenopus* oocytes. Tranilast inhibited urate transport by URAT1 and GLUT9 in a reversible and noncompetitive (mixed) manner. Tranilast failed to significantly inhibit nicotinate transport by URAT1 and the Na^+^‐dependent nicotinate transporters SMCT1 and SMCT2. Finally, tranilast also inhibited several key secretory urate transporters, with the exception of ABCG2.

Prior to studying the effect of tranilast on transporters, we first verified, optimized, and compared the urate transport characteristics of multiple urate and nicotinate transporters, given the complex interactions between these transporters with both uricosurics and antiuricosurics. Overall, we found substantial condition‐dependent differences in the activity of specific transporters, with considerable opportunity for optimization of function in oocytes. There are also substantial differences in the relative activity of the various reabsorptive and secretory transporters in *Xenopus* oocytes. We found that URAT1 can operate as a urate/nicotinate or urate/PZA exchanger, using the intracellular pool of nicotinate or PZA, and that this urate transport activity was independent of Na^+^ and putative changes in membrane potential. PZA is a metabolite of pyrazinamide, a drug used for treatment of tuberculosis and a key pharmacological probe for renal urate transport (Mandal and Mount 2015). The potent antiuricosuric action of pyrazinamide can thus be explained by trans‐activation mechanism of urate/PZA exchange by intracellular PZA (Guggino and Aronson 1985), transported into the cell by Na^+^‐dependent transporters (Manganel et al. 1985) (SMCT1 and SMCT2) where it “trans‐activates” apical exchange with luminal urate (Mandal and Mount 2015). In the absence of extracellular Cl^−^ urate, the uptake activity of URAT1 was stimulated, potentially due to the lack of cis‐inhibition by Cl^−^. In contrast to prior reports (Enomoto et al. 2002), we did not find trans‐stimulatory effects of preloaded intracellular lactate, nor did we detect cis‐inhibitory effects of 10 mM extracellular lactate on urate uptake by URAT1. We did however find that nicotinate is a substrate for URAT1, via direct measurement of [^14^C]‐nicotinate uptake, an activity of URAT1 that had not previously been established.

As observed for URAT1, the trans‐stimulatory effect of intracellular niocotinate or PZA on urate uptake by OAT10 was also significant. Notably, the absence of extracellular Cl^−^ was found to abrogate urate and nicotinate uptake activity of OAT10, which is in direct contrast to URAT1. In this respect, OAT10 is more similar to OAT1, which is also stimulated by the presence of extracellular Cl^−^ (Rizwan et al. 2007). Unlike URAT1 and OAT10, OAT4 appears to function by exchanging urate with divalent anions; in particular, the trans‐stimulatory effect of intracellular maleate on urate uptake by OAT4 was significant. With respect to basolateral urate transporters, urate transport mediated by GLUT9a was markedly stimulated by cell membrane depolarization, as reported previously (Anzai et al. 2008; Bibert et al. 2009; Witkowska et al. 2012). Urate uptake mediated by the basolateral transporter OAT3, thought to play a role in urate secretion (Eraly et al. 2008), was not trans‐stimulated by intracellular niocotinate, PZA, or *α*‐KG. The urate uptake activity of OAT3 was however cis‐inhibited by the presence of extracellular monovalent anions (10 mM), including nicotinate, PZA, and salicylate.

The monocarboxylate transporters SMCT1 and SMCT2 play important indirect roles in urate reabsorption (Mandal and Mount 2015). Although the SMCT1‐ and SMCT2‐mediated [^14^C]‐nicotinate uptake activity was primarily Na^+^‐dependent, we found significant reduction in nicotinate uptake activity of SMCT1 and SMCT2 in the absence of extracellular Cl^−^, suggesting SMCT1‐ and SMCT2‐mediated nicotinate transport is dependent on extracellular Cl^−^. This extends and confirms the prior electrophysiological data for SMCT1, showing Cl^−^‐dependence without evidence of direct Cl^−^ transport (Coady et al. 2004). Others have reported an absence of chloride dependence in SMCT1 (Miyauchi et al. 2004), which is refuted by our data and that of Coady et al. (2004).

Our studies on the effect of tranilast on multiple urate transporters revealed several novel actions of the drug. The most important observations are: (1) Tranilast effectively inhibited urate transport activities of the major reabsorptive urate transporters URAT1, GLUT9, OAT4, and OAT10; this provides an unequivocal molecular explanation for the drug's uricosuric effect; (2) Tranilast did not inhibit the nicotinate transport mediated by URAT1, SMCT1, and SMCT2, even at higher concentrations (>180 *μ*mol/L). However, OAT10‐mediated nicotinate transport was comparatively sensitive (IC_50_ of ~43.1 μmol/L) to tranilast; (3) Inhibition of URAT1 and GLUT9a by benzbromarone and probenecid was not reversible in this system, whereas tranilast interacts with these transporters in a reversible, noncompetitive manner. (4) Tranilast also inhibited the secretory transporters NPT1, OAT1, and OAT3, with no effect on ABCG2‐mediated urate efflux.

Since 90% of uric acid from glomerular filtrate is reabsorbed in the renal proximal tubule, drugs that inhibit reabsorptive urate transporters in vivo are potent uricosurics. Inhibition of secretory urate transporters by uricosurics has less effect on urate excretion, given the dominance of urate reabsorption in the kidney and the very low fractional excretion of urate in humans (Roch‐Ramel and Guisan 1999). For example, although probenecid inhibits apical OAT10/URAT1/OAT4, it also inhibits the apical secretory transporters NPT1 (Jutabha et al. 2003), NPT4 (Jutabha et al. 2010) and the basolateral secretory transporters OAT1 and OAT3; probenecid is however a widely utilized uricosuric agent (Roch‐Ramel and Guisan 1999). Notably, however, via the inhibition of secretory transporters tranilast has the capacity to affect in vivo homeostasis of urate, including compartmentalization kinetics in the central nervous system and distribution in intracellular versus extracellular compartments. Mechanistically, the inhibition of urate transport mediated by multiple urate transporters from different gene families suggests the existence of analogous interaction sites for urate and tranilast. Urate transport mediated by URAT1 was specifically inhibited by <50 *μ*mol/L tranilast, without affecting its nicotinate transport activity. This novel finding suggests distinct but likely overlapping binding sites in URAT1, one for urate and the other for nicotinate, which are sufficiently dissimilar to differentially interact with tranilast. Further studies are required to clarify interaction sites for tranilast, nicotinate, and urate in URAT1; it also remains to be determined whether other potent uricosurics differentially affect urate and nicotinate transport by URAT1.

The inhibitory action of tranilast on urate transport by URAT1 and GLUT9 was abrogated by washing oocytes, suggesting that the interaction is reversible; this differs from the behavior of benzbromarone. The results of Eadie–Hofstee linearizations of urate uptake kinetics revealed that the inhibition of URAT1 and GLUT9a‐mediated urate uptake by tranilast closely resembles a mixed noncompetitive inhibition model. Notably, the GLUT9 and URAT1 proteins exhibit minimal homology with one another. The noncompetitive inhibition (mixed) model for both urate transporters suggests that tranilast binds to a site other than the urate translocation sites, with indirect allosteric effects on urate transport.

The urate‐lowering and uricosuric effect of tranilast (Sundy and Kitt 2010) is evidently explained by the potent inhibition of multiple reabsorptive urate transporters (URAT1, OAT10, OAT4, and GLUT9). Although inhibition of any of these transporters would cause uricosuria, we postulate that GLUT9 inhibition is particularly influential in the effect of tranilast, given the more severe uricosuric phenotype for patients with GLUT9‐deficient, versus URAT1‐deficient, renal hypouricemia (Dinour et al. 2010). GLUT9 is the exclusive exit pathway for urate during reabsorption, whereas there is considerable heterogeneity for the apical entry mechanism. The urate‐lowering effect of tranilast could additionally involve inhibition of xanthine oxidase, already linked to its effect on the generation of reactive oxygen species (Miyachi et al. 1987). Notably, the two classical inhibitors of xanthine oxidase, febuxostat, and oxypurinol (the active metabolite of allopurinol) also interact with URAT1 (Iwanaga et al. 2005; Jutabha and Anzai 2014), such that additional interactions of tranilast with xanthine oxidase would not be an unexpected property for a uricosuric drug.

The previously reported effects of tranilast include antiproliferative effects (Rogosnitzky et al. 2012), activation of the nuclear aryl hydrocarbon receptor (Hu et al. 2013), inhibition of TGF‐beta (Tao et al. 2011), inhibition of cation channels (Mihara et al. 2013), mast cell stabilization (Sastre et al. 2013), and modulation of tryptophan metabolism (Munn et al. 1998). It has therapeutic uses and therapeutic potential in a wide variety of disorders, including for example diabetic nephropathy (Mifsud et al. 2003). To the extent that some of these disorders, including diabetic nephropathy (Rosolowsky et al. 2008; Ficociello et al. 2010; Doria and Krolewski 2011), are linked to hyperuricemia, it is tempting to speculate that some of the systemic therapeutic benefits of tranilast are mediated by its urate‐lowering action. In this regard, it is equally interesting that hyperuricemia is in turn linked to changes in tryptophan metabolism (Liu et al. 2011), providing indirect links between these actions of tranilast.

## Authorship Contributions

Participated in research design: Asim K. Mandal, David B. Mount. Conducted experiments: Asim K. Mandal, Andria Foster. Contributed new reagents or analytic tools: Adriana Mercado, Kambiz Zandi‐Nejad. Performed data analysis: Asim K. Mandal, David B. Mount. Wrote or contributed to the writing of the manuscript: Asim K. Mandal, David B. Mount.

## Disclosure

None declared.

## Supporting information


**Figure S1.** Tranilast inhibits urate transport activity of URAT1 in a reversible manner in *Xenopus laevis* oocytes.

**Figure S2.** Tranilast inhibits urate transport activity of GLUT9a in a reversible manner in *Xenopus laevis* oocytes.

**Figure S3.** Functional characterization of human URAT1 as a nicotinate transporter.

**Figure S4.** [^14^C]‐nicotinate transport properties of URAT1: voltage sensitivity and Na^+^‐dependence was examined by replacing extracellular NaCl by KCl or LiCl.

**Figure S5.** The 50% inhibitory concentration (IC_50_) curve of tranilast for [^14^C]‐nicotinate uptake via OAT10 in oocytes preloaded with PZA.
